# Morbidity-mortality and performance evaluation of Brahman calves from *in vitro *embryo production

**DOI:** 10.1186/1746-6148-7-79

**Published:** 2011-12-04

**Authors:** Andreza Pimenta-Oliveira, José P Oliveira-Filho, Adriano Dias, Roberto C Gonçalves

**Affiliations:** 1 School of Veterinary Medicine and Animal Science - Univ Estadual Paulista, UNESP, Botucatu, Sao Paulo, Brazil; 2 School of Medicine - Univ Estadual Paulista, UNESP, Botucatu, Sao Paulo, Brazil

## Abstract

**Background**

The use of bovine *in vitro *embryo production (IVP) increases the reproductive potential of genetically superior cows, enabling a larger scale of embryo production when compared with other biotechnologies. However, deleterious effects such as abnormal fetal growth, longer gestation period, increased birth weight, abortion, preterm birth and higher rates of neonatal mortality have been attributed to IVP. The aim of this study was to compare the influence of *in vitro *embryo production and artificial insemination (AI) on gestation length, complications with birth, birth weight, method of feeding colostrum, passive transfer of immunity, morbidity-mortality, and performance in Brahman calves.

**Results**

Whilst gestation length and birth weight were significantly increased in IVP-derived calves, no difference in weaning weight was observed between groups. The passive transfer of immunity (PT), was assessed in IVP (n = 80) and AI (n = 20) groups 24 hours after birth by determination of gamma-glutamyl transferase (GGT) and gammaglobulin activity as well as by quantification of the concentration of total protein in serum. No differences in passive transfer or incidences of dystocia and diseases at weaning were observed between groups. Birth weight, method of feeding colostrum and dystocia were not correlated with PT in either group.

**Conclusions**

In this study, *in vitro *embryo production did not affect the health status, development, or passive transfer of immunity in Brahman calves.

## Background

The use of bovine *in vitro *embryo production (IVP) has increased in Brazil over the past several years. This procedure allows for embryos to be obtained from genetically superior cows that are no longer able to produce offspring by conventional techniques or procedures. IVP increases the reproductive potential of the cows when compared with other biotechnologies such as artificial insemination (AI) [1]. However, though IVP promotes faster genetic improvement [1], it has also been associated with a number of adverse effects such as higher embryo mortality rates, abnormal fetal growth, heavier offspring, longer gestation, abortion, preterm birth, increased genetic abnormalities and high rates of neonatal mortality [2,3].

Dystocia in newborn calves can cause asphyxia and so delay onset of natural suckling, negatively affecting the passive transfer of colostral immunoglobulins [4]. Dystocia is also associated strongly with increased mortality, morbidity and with a detrimental impact on health and development from birth to weaning [5]. The failure of passive transfer causes hypogammaglobulinemia which increases the susceptibility to neonatal disease, low growth performance, and high mortality [6].

The aim of this study was to compare the influence of *in vitro *embryo production and artificial insemination (AI) on gestation length, complications with birth, birth weight, method of feeding colostrum, passive transfer of immunity, morbidity-mortality, and performance in Brahman calves.

## Methods

### Farm

The study was conducted in a purebred Brahman farm (*Bos taurus indicus*) (S: 23°04'34.7'' and W: 48°27'12.2'') located in Pardinho county in Sao Paulo, Brazil. The property comprises an area of 260 hectares, most part covered with *Brachiaria *sp. grass. The animals were raised under a semi-intensive system and were under permanent veterinary care.

### Animals

Regardless of gender, 100 Brahman calves were selected based on their method of production and divided into two groups during one calving season (i.e., six months). Eighty calves were from IVP offspring (IVP group) and 20 were produced by artificial insemination (AI group). At birth, umbilical disinfection was performed with 5% iodine. Individual animals were identified by tattoo and cow tag earrings and dewormed with 200 μg/kg of body weight (BW) of doramectin subcutaneously. The calves were kept under natural grazing conditions with mineral salt licks and water *ad libitum*. Supplemental creep feeding consisted of *Cynodon dactylon *ground hay (1% BW), corn silage (3% BW), and 1% BW of a commercial concentrate (crude protein 19%, crude fat 3%, crude fiber 12%, mineral matter 11%, calcium 1.5%, phosphorus 0.6%).

All of the calves ingested colostrum during the 6 hours from birth, irrespective if by natural suckling, bottle feeding or nasogastric tube feeding. Calves were kept with their mothers until weaning (i.e., 210 days). Over this period, clinical assessment of calves was performed and any disease treated.

The cows used in our experiment were negative for brucellosis and tuberculosis and vaccinated against infectious bovine rhinotracheitis, bovine virus diarrhea, campylobacteriosis, leptospirosis and clostridiosis. Multiparous Brangus and Simbrasil cows were used as recipients for IVP embryos. Multiparous Brahman cows were artificially inseminated and the offspring used for the AI group. The semen used for both *in vitro *embryo production and artificial insemination techniques was obtained from two Brahman sires of an independently owned semen company. Follicular aspiration as well as *in vitro *embryo production and transfer were performed by a technician from a biotechnology company. Artificial insemination was achieved by a single technician.

One month before birth, dams were placed in maternity pens with mineral salt licks and water *ad libitum *and supplemented with corn silage (3% BW) and 1% BW of a commercial concentrate (crude protein 20%, crude fat 2.5%, crude fiber 8%, mineral matter 11%, calcium 2%, phosphorus 0.5%).

### *In vitro *embryo production

Cumulus-oocyte complexes (COCs) were obtained by ovum pick-up (OPU) from Brahman donors. Each donor was subjected to one OPU session using a SSD-500 ultrasound scanner (Aloka^®^, Tokyo, Japan) with a 5 MHz sector transducer (Aloka^®^, Tokyo, Japan) attached to the transvaginal guide (Cook^® ^Medical, Bloomington, USA). Ovum pick-up was performed using an 18G needle under vacuum (500 mm Hg). The cumulus oocyte-complexes were collected into phosphate buffered saline medium (Cultilab^®^, Campinas, Brazil) with 1% fetal calf serum (FCS) and 5 IU of heparin/mL. Prior to *in vitro *maturation COCs were assessed morphologically, and only those that had a cumulus morphology quality I and II [7] were selected.

All selected COC's were *in vitro *matured in 30 × 10 mm dishes (Corning^®^, Lowell, USA) with 100 μL drops of modified TCM-199 medium with 10% FCS (Cultilab^®^, Campinas, Brazil). This medium was supplemented with 5 μg/mL of FSH (Folltropin-V^®^, Bioniche Animal Health, Belleville, Canada) and 50 μg/mL of LH (Lutropin-V^®^, Bioniche Animal Health, Belleville, Canada), 1 μg/mL of porcine estradiol, 100 μg/mL of epidermal growth factor human (EGFh), 6.25 μg/mL of insulin, 22 μg/mL of pyruvate, and gentamicin. For *in vitro *maturation the COCs were incubated for 24 h at 39°C under humidified 5% CO_2 _in air.

After maturation, oocytes were transferred to 30 × 10 mm dishes (Corning^®^, Lowell, USA) containing 100 μL Tyrode's albumin-lactate-pyruvate medium with 22 μg of pyruvate/mL, 6 mg of BSA/mL, 10 μg of heparin/mL, 20 μM of penicillamine/mL, 10 μM of hypotaurine/mL, and 2 μM of epinephrine/mL. Oocytes were inseminated with 2 × 10^6 ^sperm/mL and maintained at 39°C with 5% CO_2 _in saturated humidity for 18 to 24 h. Following insemination, putative zygotes were removed by successive pipetting. The *in vitro *culture was performed in 30 × 15 mm dishes with 100 μL Synthetic Oviduct Fluid medium [8] containing 4 mg/mL of bovine serum albumin BSA and 2% FCS. Embryos were incubated under humidified 5% CO_2_, 5% O_2 _and 90% N_2 _at 39°C. All embryos were removed from culture on day seven of development and transferred to synchronized recipients.

### Sample collection

Data were obtained regarding pregnancy technique (IVP or AI), gestation length, type of delivery (normal parturition or dystocia, use of obstetric chains or cesarean section), direct or indirect (i.e., bottle or oral-esophagic tube) colostrum feeding, and weight at birth, 30, 60, 90, 120 and 210 days after delivery. The animals were checked daily until weaning and more thoroughly when there was evidence of clinical alteration. Data on the presence and frequency of diseases were individually recorded.

Blood samples from the calves were collected via jugular puncture using a 21G needle and serum tubes, 24 hours after birth. Samples were centrifuged and serum stored in plastic tubes at -20°C until tests. The protocol was approved by the Committee of Ethics and Animal Welfare of the Univ Estadual Paulista (UNESP), School of Veterinary Medicine and Animal Science, Brazil.

### Diagnosis of failure of passive transfer (FPT)

Diagnosis of FPT was assessed by determination of gamma-glutamyl transferase (GGT) activity in serum, according to the kinetic-colorimetric method previously describe [9], with a commercial kit (Katal^®^, Belo Horizonte, Brazil) and a SB-190 automatic analyzer (Celm^®^, Barueri, Brazil) set at 405 nm length wave. Separation of the gammaglobulin protein fraction from serum was performed by electrophoresis in agarose gel (Celm^®^, Barueri, Brazil) stained with amido black (0.1%) in 5% acetic acid [10]. Samples were read using a SE-250 scanner (Celm^®^, Barueri, Brazil). Total protein was quantified by direct biuret method [11] using the automatic analyzer and a commercial kit (Katal^®^, Belo Horizonte, Brazil). FPT was considered to have occurred when the calf had at least one of the following values: total protein (< 5.0 g/dL), gammaglobulin (< 1.0 g/dL) and/or serum GGT (< 100 IU/L) [12].

### Statistical analysis

Data were analyzed by Chi-square or Fisher's exact test when variables were categorical. Due to the non-normal distribution of continuous variables, comparison between AI and IVP groups was performed using the nonparametric Mann Whitney U test. All correlations were conducted using the Spearman RHO test. The significant level of analysis was established at 5%.

## Results and discussion

Gestation length of IVP offspring was significantly longer than AI offspring (p < 0.001), with means of 294 ± 6.6 days and 287 ± 6.7 days, respectively (Table 1). Previous studies have also reported significant increases (nine days) in the gestation length of IVP calves when compared with AI calves [13]. However, this difference has not always been observed [14]. No difference in gestation length was observed between IVP and AI Gyr calves (*Bos indicus*) [15], suggesting that the effect of the IVP on the gestation length is less perceptible in zebu breeds, which intrinsically have longer gestation periods.

**Table 1:** Gestation length, delivery, feeding method, and weight from birth to weaning of the Brahman calves

Group	**Gestation length**^**1**^	Delivery		Method of feeding colostrum							
								
		Eutocic	Dystocic	Direct	Indirect	**Birth weight**^**2**^	**W30**^**3**^	W60	W90	W120	W210
**IVP**	294 ± 6.6^a^*	80% (64/80)	20% (16/80)	92.5% (74/80)	7.5% (6/80)	41 ± 5.8^a^	75 ± 13.5^a^	100 ± 14.4	130 ± 18.5	160 ± 21.4	221 ± 27.4
**AI**	287 ± 6.7^b^	95% (19/20)	5% (1/20)	95.0% (19/20)	5.0% (1/20)	38 ± 4.7^b^	67 ± 10.8^b^	94 ± 18.1	124 ± 20.2	157 ± 23.4	215 ± 32.9

^1 ^Mean gestation length (days)

^2^Mean birth weight (kg)

^3^Mean weight (kg) at 30 (W30), 60 (W60), 90 (W90), 120 (W120), and 210 (W210) days after delivery

*Different letters (a, b) indicate significant difference (P < 0.05) between IVP and AI groups

As in other studies [13-15], birth weight in our study was significantly increased (p = 0.038) in IVP when compared to AI calves, with mean values of 41 ± 5.8 kg and 38 ± 4.7 kg, respectively (Table 1). However, although the relationship between increased birth weight and longer gestation period has previously been described [16], no significant association between these variables was found in our experiment (0.166, p = 0.142 for IVP and 0.374, p = 0.104 for AI). Moreover, significant differences in gestation length were not found between IVP and AI groups. It is important to mention that though the birth weight can be influenced by the characteristics of the sire [15], the male effect was not considered in our study due to the proportional use of semen from the very same two sires in both groups. In order, to increase the efficiency of IVP [17] we supplemented the bovine embryo culture medium with FCS. Long-term effects of medium composition on fetal development have been observed such as increased birth weight after culture in media containing serum [14,18]. However, other authors [19] did not observe influence in birth weight among IVP calves regardless the use of embryo culture with or without FCS.

Males were heavier than females at birth in a previous work [20]. In our study males were heavier at birth in both groups. Males (43.5 ± 4 kg) and females (39 ± 6.2 kg) from the IVP group were heavier than the males (41 ± 2.9 kg) and females (36 ± 4.1 kg) from the AI group. These results agree with other studies [13-15], which compared body weight between IVP and AI offspring. However, breed of the mother could be a factor affecting offspring birth weight. It is known that Brahman cows limit fetal growth during the last third of gestation when compared with Charolais cows [21]. This restriction seems to be a consequence of decreased weight of placentomas and reduced uterine blood flow in Brahman cows [22]. In contrast to the results from these authors [21,22] the breed of the recipient cow was not incorporated as a factor influencing birth weight [23]. These authors attribute the increased birth weight of IVP offspring to factors such as the use of multiparous recipients, sex of the calf, handling conditions and intrinsic characteristics of the IVP technique e.g., use of frozen embryos. Thus, as all cows in our study were multiparous and submitted to the same procedures under the same handling conditions, we strongly believe that the breed of the recipient cows in both IVP (mixed zebu) and AI (pure zebu) groups did not influence the birth weight of the calves. Moreover, the use of different breeds as embryo receptors was not considered in the assessment of birth weight, gestation length, and development of calves produced either for IVP or AI in previous studies [24].

IVP individuals may have abnormal placental and/or fetal development, causing the so called large offspring syndrome, characterized by excessively large calves at birth, high perinatal mortality, hydrallantois and increased number of abortion [18]. Calves with large offspring syndrome were not present in our study. Even so, 6.3% (5/80) of the IVP-derived calves displayed a mean birth weight 30% heavier than the mean AI calf weight. The same pattern was found at day 30 i.e., IVP calves were significantly heavier than AI calves (p = 0.017) (Table 1). However there were no significant differences in mean weight between groups at 60, 90, 120 and 210 days of age (i.e., weaning) which demonstrates the effect of feeding on offspring development (Table 1).

Weight and size at birth, associated with the specific characteristics of the recipients and embryos, as well as with the number of births, were the main factors causing dystocia in Nelore recipients [25]. Other authors reported more dystocia in IVP than AI calves (62% and 10%, respectively) [14]. However, the increased birth weight of IVP-derived offspring (n = 26) did not provoke delivery problems when compared with AI calves (n = 32) [15]. In the same study, only one case of dystocia was present in a 35 kg IVP-derived calf, whilst the heaviest animal was a 37 kg IVP female [15]. It is therefore, possible that the low rate of dystocia has been a consequence of the relatively lower birth weight of IVP Gyr calves compared with other breeds, e.g., Nelore [15]. Dystocia was only observed in *in vitro *and *in vivo *derived calves weighing more than 40 kg [25].

Despite observing dystocia in 20% (16/80) of IVP births, i.e., one cesarean section and 15 births using obstetric chains; and in 5% (1/20) of the AI calves, no significant differences were identified between groups (p = 0.096) (Table 1). Fourteen of the 16 IVP calves with dystocia had birth weight above the mean value for this group ranging between 41 and 56 kg. Therefore, whilst birth weight contributes to the occurrence of dystocia, other factors must also be evaluated at mating and in the selection of recipient cows [25].

Dystocia may result in trauma to the newborn affecting its disposition to nurse. Although normal neonates may show mild acidosis, the intense and prolonged delivery contractions and the inability to breathe provoke an acid-base imbalance in calves with dystocia [26]. Long and laborious delivery, e.g., more than 4 hours, can cause fetal hypoxia, tongue edema, cerebral edema [4], and decreased physical activity leading to a longer period from parturition until the calf stands and nurses colostrum by itself [27]. In fact, this entire clinical picture may diminish or completely cease colostrum absorption [28]. In our study, six calves from the IVP group (bottle n = 4, esophageal tube n = 2) and one from the AI group (esophageal tube n = 1) were forced-fed colostrum. No significant differences were found between groups regarding the method of feeding colostrum (p > 0.05) (Table 1). Three of the seven animals (two IVP and one AI calf) that were fed colostrum through non-natural methods suffered dystocia. Still, association between dystocia and non-natural suckling of colostrum was not observed (p = 0.110).

Since maternal immunoglobulin transfer through the placenta does not occur in cattle, the absorption of colostral antibodies is critical to promote the passive immunity during neonatal life [29]. In order to improve passive transfer, colostrum was given either directly or indirectly during the first 6 hours post-delivery in this study.

At least one of the three analyzed variables supposes to be below the normal limits for FPT diagnosis, i.e., total protein (< 5.0 g/dL), gammaglobulin (< 1.0 g/dL) and/or serum GGT (< 100 IU/L). Failure of passive transfer was detected in 10% of the IVP offspring (8/80) and 20% of the AI offspring (4/20) without significant difference between groups. Only one AI-derived calf had lower GGT (22.3 IU/L), total protein (4.7 g/dL) and gammaglobulin (0.7 g/dL). Nevertheless, this animal did not experience disease and had a weaning weight of 208 kg, very close to the mean weight of the group.

Though passive transfer is essential for calf survival, several other factors may influence life and performance of the individual [6,30,31]. As in our study, *Bos taurus *IVP-derived calves absorb large amounts of colostral immunoglobulins in order to acquire adequate passive immunity [32,33]. In agreement with our study, Jacobsen et al. also reported no significant differences in IgG concentration among IVP and AI offspring [33]. The authors concluded that the low viability of IVP calves is probably unrelated to the ability and/or efficiency of the calf to obtain passive immunity through colostrum [33].

The birth weight, method of feeding colostrum and dystocia were not related with FPT in any of the groups within the current study. Some studies have shown correlations between the increase in FPT and dystocia [34,35]; however, only one of the IVP animals with dystocia (1/16) in the present study suffered FPT (gamaglobulin = 0.37 g/dL). Thereby, the low occurrence of FPT in calves with dystocia in our study agree with other authors, who reported no significant differences in serum immunoglobulins among normal and distocic calves [36], as well as no significant differences in serum total protein and IgG after normal or assisted delivery [37]. However, longer delivery (irrespective if normal or assisted) led to low serum levels of IgG, probably because the longer period until nurse and therefore less colostrum ingested [37].

In general, the low occurrence of FPT in both groups in this study was probably associated with the immediate and comprehensive assistance provided at delivery, thus minimizing the risk of fetal hypoxia and increasing the rate of feeding with colostrum during the first 6 hours after birth. Therefore, the FPT seems to be more related to fail in ingestion of colostrum by the calf rather than to a defective absorption of macromolecules through the intestinal epithelium [37].

At least one disease was present in 40% (32/80) and 50% (10/20) of the IVP and AI groups respectively (p = 0.42) (Table 2). Diarrhea had the highest prevalence i.e., 26 (32.5%) of the IVP calves and seven (35%) of the AI calves, with no statistical difference between groups (p = 0.83) (Table 2). Several authors points out the diarrheic syndrome as one of the most important diseases in neonate calves in Brazil [23,38,39] and other countries [40]. As previously observed [36], most cases of diarrhea were between the 4th and 6th week from birth.

**Table 2:** Diseases and death frequencies of the Brahman calves derived from IVP and AI

Group	Diseases	Diarrhea	**UD**^**1**^	Tick fever	Corneal ulcer	Dermatophilosis	**BCP**^**2**^	Death
**IVP**	40% (32/80)	32.5% (26/80)	11.3% (9/80)	5% (4/80)	5% (4/80)	1.2% (1/80)	__^a^*	3.8% (3/80)
**AI**	50% (10/20)	35% (7/20)	5% (1/20)	5% (1/20)	__	__	10%^b ^(2/20)	5% (1/20)

^1^Umbilical diseases

^2^Bronchopneumonia

*Different letters (a, b) indicate significant difference (P < 0.05) between IVP and AI groups

Several risk factors are related to the occurrence of diarrhea, such as excessive exposure to ammonia in closed or airless environments [41] and exposure to enteropathogens [36]. Feeding management, (i.e., nurse [39], milk substitute feeding [41,42], early use of silage in calves' diet [41]) or any other factor interfering in the passive transfer [41] are critical for the outbreak of diarrhea.

We did not observe association between diarrhea and FPT (p = 0.757). As previously described [12] it is fearful to claim that a neonate is less susceptible to suffer a specific infectious disease based exclusively on the concentration of serum immunoglobulins or indirect parameters. Therefore, other factors related to the quality of the acquired immunity, such as load of pathogens and ingestion of pathogen-specific immunoglobulins, probably justify the mild occurrence of diarrhea in both groups. On the other hand, three IVP calves (3/80, 3.8%) died (Table 2) due to diarrhea and concomitant diseases i.e., omphalitis and bovine babesiosis and anaplasmosis (tick fever). One AI calf also died due to pneumonia (1/20, 5%) (Table 2). No significant differences between the two groups were found for mortality. The three IVP animals which died presented FPT and a significant association between FPT and mortality was observed in this group (p = 0.025). The adequate colostrum feeding [42], permanent veterinary care, and good environmental and nutritional conditions, probably played a role in the low mortality rate, as diarrhea, umbilical diseases e.g., omphalitis, omphalophlebitis, umbilical myasis, and persistent urachus, are very common in calves [12,25,38]. Recently, researchers compared IVP and AI techniques, regarding the occurrence of umbilical diseases and observed significant correlation between persistent urachus and IVP [43]. The use of 5% iodine solution for 5 days and subcutaneous doramectin is considered a good practice to prevent umbilical disease in beef cattle [38]. No significant difference was identified regarding umbilical disease between groups (p = 0.42) (Table 2). The increased number of umbilical disease observed in the IVP group (n = 9) compared with the AI group (n = 1) may be caused by congenital anomalies or failure of umbilical cord retraction [43]. In our study, omphalitis and persistent urachus were the second most evident diseases in the IVP group (9/80), but no association between these abnormalities and FPT was found. Other diseases were also diagnosed in the IVP group as follows: bovine babesiosis and anaplasmosis (4/80), corneal ulcer (4/80), and dermatophilosis (1/80). In the AI group, bronchopneumonia (2/20), and bovine babesiosis/anaplasmosis (1/20) were detected. Statistical difference was observed regarding the presence of bronchopneumonia (p = 0.038) (AI > IVP group) which affected 10% of the calves of the AI group (Table 2).

In Brazil, livestock production is characterized by large-scale extensive operations under grazing conditions mainly at north and central west of the country. Although the main reproductive strategies are artificial insemination and natural matting, the use of IVP increases each year in animals of high genetic value. With the objective of diminishing the influence of environmental factors, our experiment was performed through a single farm during one calving season. Moreover, this property actually represents an elite livestock Brazilian farm using IVP as the main biotechnology for reproduction and the number of animals used in this experiment accurately reflects the characteristics of the livestock segment in this country. Other authors [15,24] obtained similar results than those in our study regarding gestation length, birth weight and calf development using a slightly lower number of animals. Thus, we emphasize that under these conditions the results from our experiment are reliable and did not suffered the influence of size of the groups.

## Conclusions

Based on our results we can conclude that gestation period and birth weight were higher for the IVP offspring. However, weaning weight was similar between groups. The occurrence of dystocia, assistance for colostrum feeding, FPT and disease in calves were similar from birth to weaning in both groups. Birth weight, method of feeding colostrum and dystocia were not associated with FPT. Under these conditions, we can say that IVP technique did not have influence on health status and performance of Brahman calves or interfered with acquisition of passive immunity.

## Competing interests

The authors declare that they have no competing interests.

## Authors' contributions

APO and JPOF performed the clinical examination, laboratorial analysis, reviewed the literature and prepared the manuscript. AD performed the statistical analysis and prepared the manuscript. RCG reviewed the literature and prepared the manuscript. All authors read and approved the final manuscript.

## Authors' information

APO, JPOF and RCG are from School of Veterinary Medicine and Animal Science - Univ Estadual Paulista, UNESP, Botucatu, Sao Paulo, Brazil. AD is from School of Medicine of the UNESP, Botucatu, Sao Paulo, Brazil.
